# Effect of Cd–Zn compound contamination on the physiological response of broad bean and aphids

**DOI:** 10.3389/fphys.2025.1533241

**Published:** 2025-02-05

**Authors:** Liya Chen, Sijing Wan, Qintian Shen, Keting Zhao, Yanlan He, Yexin Xie, Shiyu Tao, Shuchang Zheng, Yi Zhang, Shigui Wang, Bin Tang, Yan Li

**Affiliations:** ^1^ College of Life and Environmental Sciences, Hangzhou Normal University, Hangzhou, Zhejiang, China; ^2^ Department of School of Basic Medical Sciences, Tianjin Medical University, Tianjin, China

**Keywords:** heavy metal pollution, Cd–Zn mixed stress, food chain, synergistic inhibition, cumulative proline stress resistance, vitellogenin

## Abstract

**Introduction:**

The heavy metal elements cadmium (Cd) and zinc (Zn) often coexist in nature, making the environmental media more prone to compound pollution. However, research on the toxic effect of the Cd–Zn combination is still lacking, and the underlying toxic mechanisms remain unclear.

**Methods:**

Therefore, in this experiment, we established four treatment groups with different ratios of Cd–Zn compound stress for the broad bean, *Vicia faba* L., and aphids, *Megoura crassicauda*, to explore the growth and physiological adaptation mechanisms under different levels of mixed heavy metal stress.

**Results:**

By measuring the germination rate, seedling height, and chlorophyll content of broad beans, we found that Cd–Zn-mixed stress has a synergistic inhibitory effect on the growth and development of broad beans. Cd and Zn can be transferred through the food chain, while broad beans can resist complex stress by regulating the content of total soluble sugars and photosynthetic pigments in the body, as well as accumulating proline. In addition, in the first generation of adult aphids, treatment with Cd (12.5 mg/kg) + Zn (100 mg/kg) significantly affected the expression of trehalase (*TRE*) and trehalose-6-phosphate synthase (*TPS*) genes and influenced the carbohydrate content and trehalase activity in the aphids.

**Discussion:**

The number of offspring produced by the second-generation aphids was significantly reduced under mixed heavy metal treatment, but it was not caused by changes in the vitellogenin (Vg) content. These related results provide new avenues for further exploration of plant responses to mixed heavy metal stress, pest control, and management of heavy metal pollution.

## 1 Introduction

With the rapid development of industries and the extensive use of pesticides and fertilizers, environmental elements such as soil, water, and air—essential for the survival of humans and various organisms—have been severely polluted by heavy metals (Xiao, 2009; Zeng, 2022). According to statistics, the area of arable land in China has reached 1,278,666.6 km^2^, while the area of heavy metal-polluted soil is as high as 3.33 million hm^2^, with mercury (Hg), lead (Pb), zinc (Zn), cadmium (Cd), and other factors exacerbating heavy metal pollution in arable land and soil (Wang, 2023). Farmland is a crucial component of land that plays a vital role in agricultural development and the national economy. Therefore, soil heavy metal pollution has inevitably become a global problem that urgently needs to be solved (Wang et al., 2024).

When plants are contaminated with Cd, cell division slows down, chloroplasts are severely damaged, respiration and photosynthesis are negatively affected, the transpiration rate gradually decreases, and growth and development are inhibited (El Rasafi et al., 2022; Zhang and Lu, 2024). The content of heavy metals such as Cu, Pb, and Zn in garden plants in the Yunnan region and the same organs of different garden plants follow the order of Zn > Pb > Cu (Fan et al., 2016). Perhaps, because Zn and Cu are essential elements for plant growth and plants have a much greater demand for Zn than Cu, physiological processes such as chlorophyll synthesis and photosynthesis require Zn (Yadegari, 2017; Olivari et al., 2020).

A typical stress response of plants under abiotic stress is a sharp increase in soluble sugar content, especially glucose, sucrose, and fructose (Couée et al., 2006). Pb and Zn stress can reduce the activity of castor sugar metabolism enzymes (Gao, 2019). Meanwhile, due to the influence of heavy metals on plant amino acid metabolism, ecological plants with higher levels of proline have a higher tolerance to heavy metals such as Cu and Zn (Xu, 2018). However, not all plants accumulate proline under abiotic stress conditions; there is no significant correlation between the degree of proline accumulation in some plants and their stress tolerance (Hu and Xu, 2014). Zn is an essential component of carbonic anhydrase, a key enzymein plant chloroplasts that catalyzes the fixation of carbon dioxide. Therefore, appropriately supplementing Zn can increase the chlorophyll content in plant leaves (Xu et al., 2003). However, high concentrations of Zn can exhibit inhibitory effects on plant photosynthesis and growth and development (Wang et al., 2021). When the Cd concentration was 8.9 × 10^−5^ mg L^−1^, the photosynthetic pigment content of *Halophila ovalis* was significantly inhibited (Ralph and Burchett, 1998). However, heavy metals such as Zn and Cd often coexist in nature, and the effects of combined pollution vary among different plants. Under the same Cd concentration treatment, the trend of chlorophyll decline is more significant when exogenous Zn is added to *Lycopersicon esculentum*, with all treatment groups showing lower chlorophyll levels than the control group (Zhao et al., 2020).

As is well known, the production of eggs is one of the most energy-demanding events in the adult life of female insects (Lu et al., 2019), and trehalose is the major circulating sugar used in oocyte growth, accounting for 80%–90% of the total sugar in insect hemolymph (Thompson, 2003). Trehalose metabolism not only regulates the biosynthesis of chitin in insects, thereby affecting their growth and development (Merzendorfer and Zimoch, 2003; Chen et al., 2010), but also participates in the uptake of vitellogenin (Vg) by oocytes, indirectly affecting their development (Wang et al., 2020). In eukaryotes, trehalose is only synthesized through the trehalose synthase/trehalose-6-phosphatase pathway (TPS/TPP) (Avonce et al., 2006). Trehalase (TRE) is the only enzyme that can irreversibly hydrolyze trehalose into glucose, providing energy for insect growth, development, and activity (Luo et al., 2022; Wang, 2022). In addition, heavy metal stress can affect insulin biosynthesis and secretion by inhibiting the expression of key genes in the insulin signaling pathway, thereby affecting trehalose metabolism in adult *Spodoptera litura* (Yang, 2021a).

Insect Vg is a high-molecular weight glycolipid complex protein composed of approximately 1%–14% carbohydrates, 6%–16% lipids, and 84% amino acids (Hiremath and Lehtoma, 1997; Tufail and Takeda, 2008). It can undergo a series of complex biochemical processes and precipitate yolk protein (YP) in crystal form (Matozzo et al., 2008; Tufail and Takeda, 2009), providing nutrients for oocyte maturation and embryonic development. Under the stress of 5,000 mg/kg Pb, the expression of the *Vg* gene in female *Musca domestica* on the first day of emergence is significantly downregulated (Liu, 2023). In addition, studies have shown that under Pb stress, the relative expression of the female *Vg* gene was downregulated in 5th-instar larvae and adults of *Spodoptera exigua* (Qian, 2016).

*Vicia faba* L. is an important edible legume crop that combines food, vegetables, feed, medicine, and green manure in China (Zhang et al., 2018). It is rich in nutrients and serves as an important crop for land use and intercropping, playing a vitalrole in the adjustment of planting structures. It is also one of the characteristic economic crops in northwest China (Li et al., 2015). However, due to its periodic parthenogenesis, short generation cycle, and strong reproductive and dispersal abilities, the population of *Megoura crassicauda* can rapidly grow in the short term (Feng, 2015), causing huge losses to leguminous crops and forage (Wu et al., 2015). Consumers at all levels can transfer and accumulate heavy metals in the food chain through predation relationships (Di, 2017). For example, after treating soil with heavy metal solution, higher levels of heavy metals were detected in eggplants, tomatoes, broad beans, mealybugs, aphids, and ladybugs than in the control group (Wang et al., 2017a; Butt et al., 2018; Naikoo et al., 2021; Li et al., 2024; Yin et al., 2024), indicating that heavy metals can flow and accumulate through the food chain. However, a large amount of research has only focused on the treatment of single heavy metal element stress, neglecting the fact that in most practical situations, it is a combination of multiple heavy metal elements (Yang, 2021b). In addition, most heavy metal stress cultures start from seedlings (Li et al., 2022; Hassani et al., 2023). Furthermore, Cd and Zn belong to the same element family, with similar chemical and environmental characteristics (Garg and Kaur, 2013), and studies have observed that Zn pollution and Cd pollution in soil could occur simultaneously (Wang et al., 2017b). Therefore, in this study, Zn and Cd were selected as stress factors, and four treatment groups were set up with single Cd and Cd–Zn with different ratios of compound stress. Heavy metal element compound exposure was applied to broad bean seeds to explore the growth and physiological adaptation mechanisms of broad beans under different levels of mixed heavy metal stress and the impact of this stress on aphids through the food chain.

## 2 Materials and methods

### 2.1 Test plants and insect sources

The type of broad beans (*V. faba*) used in this experiment was Qingchan No.14, and the feeding conditions were as follows: temperature, 19°C ± 1°C; humidity, 70% ± 5%; and photoperiod, 14 L:10 D. The aphid species was *M. crassicauda*, and the breeding conditions were the same as those of broad beans.

### 2.2 Design of experiments

Referring to the research report by Wang et al. (2017a), solutions of Cd^2+^12.5, Cd^2+^12.5+Zn^2+^50, Cd^2+^12.5+Zn^2+^100, and Cd^2+^12.5+Zn^2+^150 mg/kg were set as experimental treatment groups. Mixed solutions of CdCl_2_ and ZnSO_4_ were prepared in different ratios; the broad bean seeds were soaked for 24 h and then planted in soil (nutrient soil: vermiculite: perlite = 12:4:2). According to the growth requirements of broad beans, 400 mL of the corresponding mixed solution was watered every 3 days. The seeds were soaked in tap water (0 mg/kg, CK group) and watered as a control group. On day 25, broad beans were collected from three sampling points near 30°17′50.84″N and 119°59′42.9″E. Three samples per material were collected from each location. The soil attached to the surface of broad bean roots was rinsed with tap water during the collection process. The components of broad beans were separated, dried, and ground into powder for subsequent research.

According to the growth status of broad beans, on day 10 of planting, the aphids that had not been treated with heavy metals should be moved onto the broad bean seedlings. After 10 days of infection, the aphids (set as the first generation) were collected and transferred to the corresponding mixed solution-treated broad bean seedlings. After 10 days of infection with the first generation of adult aphids, the adult aphids produced by the first generation (second generation) were collected and transferred to the corresponding mixed solution-treated broad bean seedlings. This process was continued, infecting subsequent generations until the third-generation aphids treated with heavy metals were collected.

### 2.3 Determination of the germination rate and seedling height of broad beans

A total of 42–45 broad beans should be planted in each group of soil, with four replicates for each soil type. The germination of broad beans is observed and recorded in each group of soil on days 7, 9, 11, and 13, and the germination rate is calculated up to the *n*th day (n = 7, 9, 11, and 13). In addition, the seedling height of broad bean seedlings is measured after 9, 11, 13, and 15 days of planting in these five soils: 10 broad bean plants are randomly selected from each soil group for measurement, and this process is repeated six times for each soil type.

### 2.4 Determination of the heavy metal content in broad beans

A sample of 0.5 g of broad bean roots, stems, and leaves was weighed and placed into a digestion tank; a measure of 4 mL of nitric acid was added, pre-oxidized at 80°C for 1 h, and placed in a microwave digestion instrument for programmed digestion. After digestion, the first-class water was transferred to a 50-mL volumetric flask and made up to volume. Inductively coupled plasma mass spectrometry (ICP-MS) was then used to detect the heavy metal content in each experimental material.

### 2.5 Determination of soluble total sugar and proline (pro) and chlorophyll content in broad beans

The roots, stems, and leaves of broad beans were collected on day 25 after planting. They were baked in an oven at a temperature of 110°C for 15 min and then reduced to 70°C overnight. Dried broad bean materials were ground and used to determine the total soluble sugar content.

The Pro content was determined using the Nanjing Jiancheng Reagent Kit (Proline Assay Kit, Nanjing, China). Please refer to the manufacturer’s instructions for the methods of determination.

Leaves (the second pair from top to bottom, in the same position as the fresh beans and irrigated with a mixed solution of different ratios over 25 days) were used for the determination of chlorophyll content. The *Guide to Modern Plant Physiology Experiments* was used for specific operations and calculation methods.

### 2.6 Determination of carbohydrate content, trehalase activity, and gene expression levels in aphids

The carbohydrate content and trehalase activity in aphids were determined using the method described by Zhang et al. (2017).

As described in Section 2.2, the first- to third-generation adult aphids were collected, with 10 adult aphids per biological replicate, and three biological replicates were included. According to the manufacturer’s instructions, an RNAiso Plus Kit (Invitrogen, Carlsbad, CA, United States) was used to extract the total RNA of insects, then 1% agarose gel was prepared for electrophoresis to detect the integrity of RNA, and NanoDrop™ 2000 (Waltham, MA, United States) was used to determine the purity and concentration of RNA. cDNA first strand was synthesized according to the instructions of the reverse transcription kit, PrimeScript™RT Reagent Kit, with gDNA Eraser (TaKaRa, Kyoto, Japan). The RT-qPCR reaction system (TaKaRa TB Green®Premix Ex Taq™) was used as follows: 1 µL of cDNA, 5 µL of TB Green, 0.4 µL of forward primer, 0.4 µL of reverse primer, and 3.2 µL of ddH_2_O. The RT-qPCR reaction procedure was as follows: pre-denaturation at 95°C for 30 s, 40 cycles of 95°C for 5 s, and 55°C–60°C for 30 s. *M. crassicauda* actin-mRNA was used as an internal control, and the primer sequences are shown in Supplementary Table S1. The RT-qPCR data were analyzed using the 2^−ΔΔCT^ method (Livak and Schmittgen, 2001).

### 2.7 Aphid fecundity determination

As described in Section 2.2, on day 10 after planting broad beans in the soil, the adult aphids that had not been infected with heavy metals were moved onto the broad bean seedlings. After 10 days of infection, aphids (F1) were collected and transferred to the corresponding mixed solution-treated broad bean seedlings. Aphid production was measured every 24 h for a total of 7 days. Aphid production in the three batches of female aphids was counted. Eight biological replicates were included for each group.

### 2.8 Statistical analysis

SPSS Statistics 26 software was used to analyze the data. Tukey’s test of one-way ANOVA was performed to test the significance of differences among treatments. Finally, software GraphPad 8.0 and Office 2021 were used to draw charts. The result graphs were represented by the mean ± standard error (SE) of independent replicates. The analysis results of Tukey’s method are represented by letters, and different letters indicate significant differences (*P* < 0.05) between different treatment groups of the same sample.

## 3 Results and analysis

### 3.1 Germination rate, seedling height, and chlorophyll content in the leaves of broad beans after Cd–Zn mixed intervention

**FIGURE 1:**
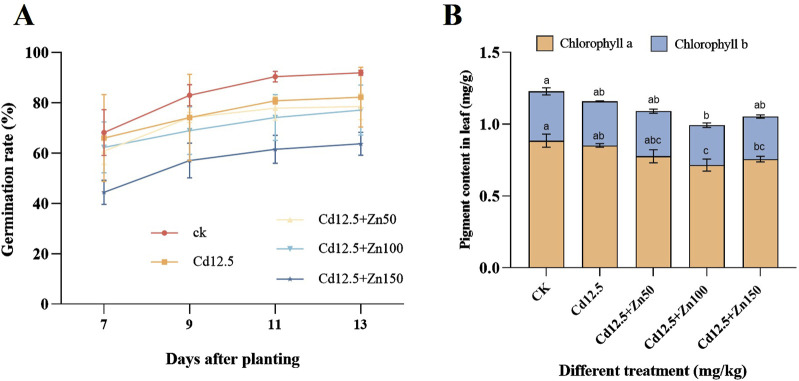
Changes in the germination rate **(A)** after days 7, 9, 11, and 13 and chlorophyll content **(B)** after day 25 of broad bean planting under different stress. The data are presented as the mean ± SE (n = 10 and N = 3). Capital letters indicate significant differences between the treatment groups for chlorophyll a, while lowercase letters indicate significant differences between the treatment groups for chlorophyll b (Tukey’s method, *P* < 0.05).

It can be observed that the germination rate of broad beans in different treatments rapidly increases from 7 to 11 days, and the increase is relatively flat from 11 to 13 days. In addition, with the increase in the Zn concentration, the germination rate of broad beans decreased and was lower than the blank control level (Figure 1A). Chlorophyll a, b, and a + b in broad bean leaves of different treatment groups were significantly lower than those in the CK group, and the chlorophyll content in broad bean leaves of the Cd12.5 + Zn100 mg/kg group was significantly lower than that of the other groups (Figure 1B). According to Table 1, on days 11,13, and 15, the seedling height of the Cd12.5 + Zn150 mg/kg group was consistently significantly lower than that of the other groups, while on day 13, the seedling height in the Cd12.5 mg/kg and Cd12.5 + Zn100 mg/kg groups was significantly higher than that in the CK group.

**TABLE 1:** Height of broad beans in different groups.

Height of broad beans (cm)					
Days after planting	0 mg/kg	Cd12.5 mg/kg	Cd12.5 +Zn50 mg/kg	Cd12.5 +Zn100 mg/kg	Cd12.5 +Zn150 mg/kg
9	3.66 + 0.35a	4.14 + 0.59a	3.63 + 0.48a	3.81 + 0.33a	3.56 + 0.45a
11	8.06 + 0.46a	8.06 + 0.46a	8.28 + 0.66a	7.31 + 0.44b	6.35 + 0.29c
13	12.95 + 0.55b	13.72 + 0.77a	13.06 + 0.45b	13.73 + 0.78a	10.97 + 0.64c
15	17.21 + 0.91a	15.49 + 0.56c	16.48 + 0.70b	15.59 + 0.70c	14.17 + 0.61d

Note: The difference between different letters in this table is the comparison between different heavy metal treatments at the same time; the unit is cm; each point is the mean ± SE of three replicates.

### 3.2 Heavy metal content in broad bean roots, stems, and leaves

**FIGURE 2:**
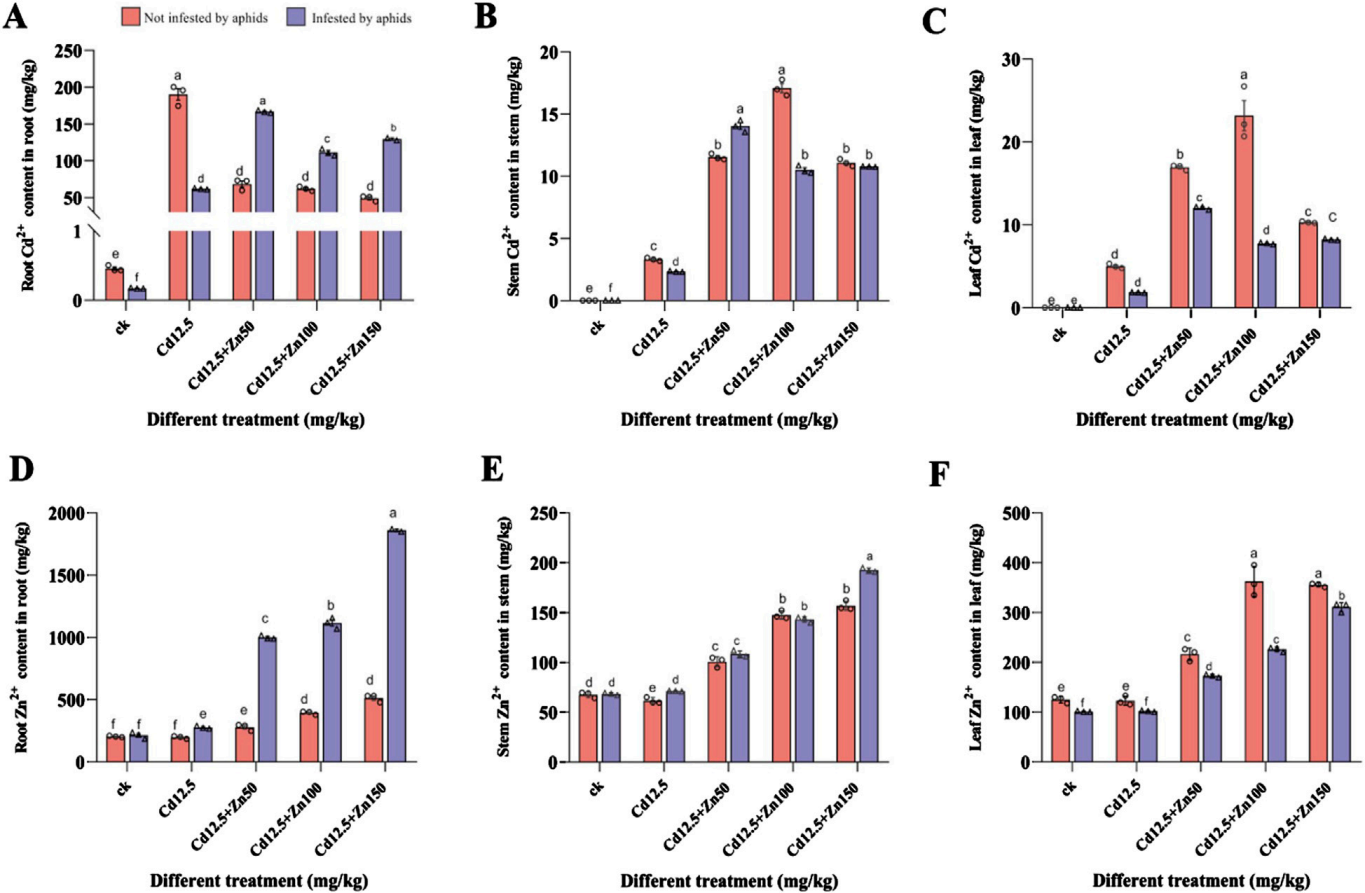
Cd^2+^ and Zn^2+^ content in the roots **(A, D)**, stems **(B, E)**, and leaves **(C, F)** of broad beans. The data are presented as the mean ± SE (n = 3 and N = 3). Bars with different letters indicate significant differences between different treatment groups of the same sample (Tukey’s method, *P* < 0.05).

In the broad bean roots, except for the CK and Cd12.5 mg/kg groups, the Cd content in plants infested by aphids was significantly higher than that in the non-infested group (Figure 2A). Moreover, only the Zn content in the broad bean roots from the non-infested plants in the CK group was not significantly different from that in the infested group, while the other groups showed extremely significant differences (Figure 2D). In the broad bean stems, the Cd content in the Cd12.5 mg/kg, Cd12.5 + Zn50 mg/kg, and Cd12.5 + Zn100 mg/kg groups, which were not infested by aphids, showed extremely significant differences compared to the group infested by aphids (Figure 2B), while the Zn content infested by aphids in the Cd12.5 + Zn150 mg/kg group was significantly higher than that in the non-infested group (Figure 2E). In the broad bean leaves, the Cd content in the Cd12.5 + Zn50 mg/kg and Cd12.5 + Zn100 mg/kg groups that were not infested by aphids was significantly higher than that in the group infested by aphids (Figure 2C). The Zn content in broad bean leaves not infected by aphids showed an extremely significant increase in the Cd12.5 + Zn100 mg/kg group, while in other groups, it showed a significant increase (Figure 2F).

### 3.3 Total soluble sugars and pro content in broad bean roots, stems, and leaves

**FIGURE 3:**
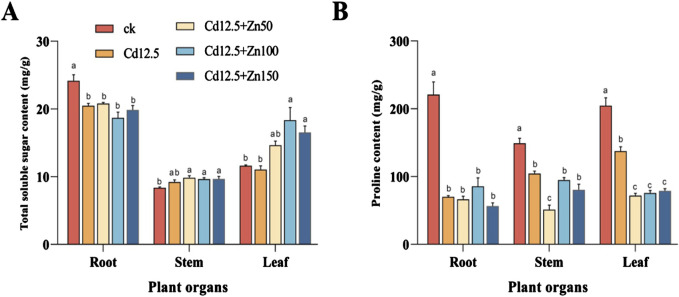
Changes in the content of total soluble sugar and proline in broad bean roots, stems, and leaves after 25 days under different stress. Total soluble sugar content of broad bean tissue in different groups **(A)**. Proline content of broad bean tissue in different groups **(B)**. The data are presented as the mean ± SE (n = 10, N = 3). Tap water was used in the control group. Bars with different letters indicate significant differences between different treatment groups of the same sample (Tukey’s method, *P* < 0.05).

In the root of broad beans, the total soluble sugar content and Pro content of different treatment groups were significantly lower than those of the CK group. In the stem of broad beans, the total soluble sugar content of different treatment groups was significantly higher than that of the CK group, while the Pro content was significantly lower than that of the CK group. Among them, the Pro content in the Cd12.5 + Zn50 mg/kg group was significantly lower than that in the other treatment groups. In the broad bean leaves, the total soluble sugar content of the three Cd–Zn composite stress treatment groups was significantly higher than that of the CK and Cd12.5 mg/kg groups, but the Pro content of the Cd12.5 mg/kg group was significantly higher than that of the other three Cd–Zn composite stress treatment groups. However, the proline content of the Cd12.5 mg/kg group was still significantly lower than that of the CK group (Figure 3).

### 3.4 Aphid carbohydrate content and trehalase activity

**FIGURE 4:**
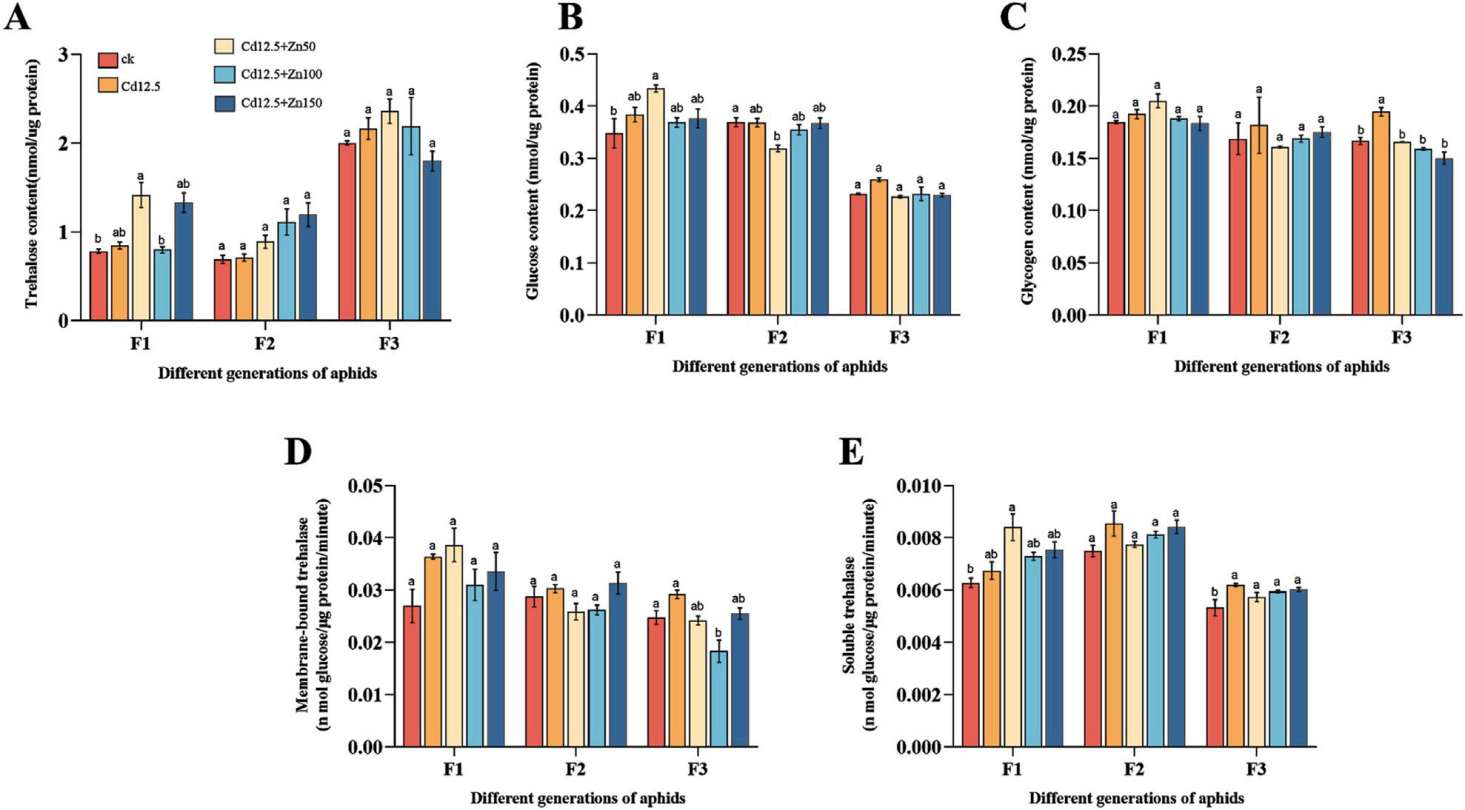
Changes in the carbohydrate content in different generations of aphids, following 21 days of different stress conditions. **(A)** Changes in the trehalose content during stress, **(B)** changes in the glucose content under different stress conditions, **(C)** changes in the glycogen content during stress, **(D)** changes in the membrane-bound trehalase activity under different stress conditions, and **(E)** changes in soluble trehalase activity under different stress conditions. Bars represent the mean ± SE of three replicate experiments (n = 10 and N = 3). Tap water was used in the control group. Bars with different letters indicate significant differences between different treatment groups of the same sample (Tukey’s method, *P* < 0.05).

In different groups of F1 aphids, the trehalose content in the control and Cd12.5 + Zn100 mg/kg groups was significantly lower than that in the other groups (Figure 4A), and the glucose content and soluble trehalase activity in the control group were significantly lower than those in the other groups (Figures 4B, E). However, there was no significant difference in the glycogen content and membrane-bound trehalase activity in the aphids of each group (Figures 4C, D). In addition, there was no significant difference in the trehalose content in the F2 and F3 generations of aphids between different groups (Figure 4A). In the F2 adult aphids, the glycogen content and two types of trehalase activity in each group of aphids were not significantly different, but the glucose content in the Cd12.5 + Zn50 mg/kg group was significantly lower than that in the other groups (Figure 4B). There was no significant difference in the glucose content in the F3 aphids (Figure 4B), while the glycogen content in the Cd12.5 mg/kg group was significantly higher than that in the other groups (Figure 4C). The soluble trehalase activity in the treatments was significantly higher than that in the control group, while the membrane-bound trehalase activity in the Cd12.5 + Zn100 mg/kg group was significantly lower than that in the other groups (Figures 4D, E).

### 3.5 Expression levels of genes related to trehalose metabolism in aphids

**FIGURE 5:**
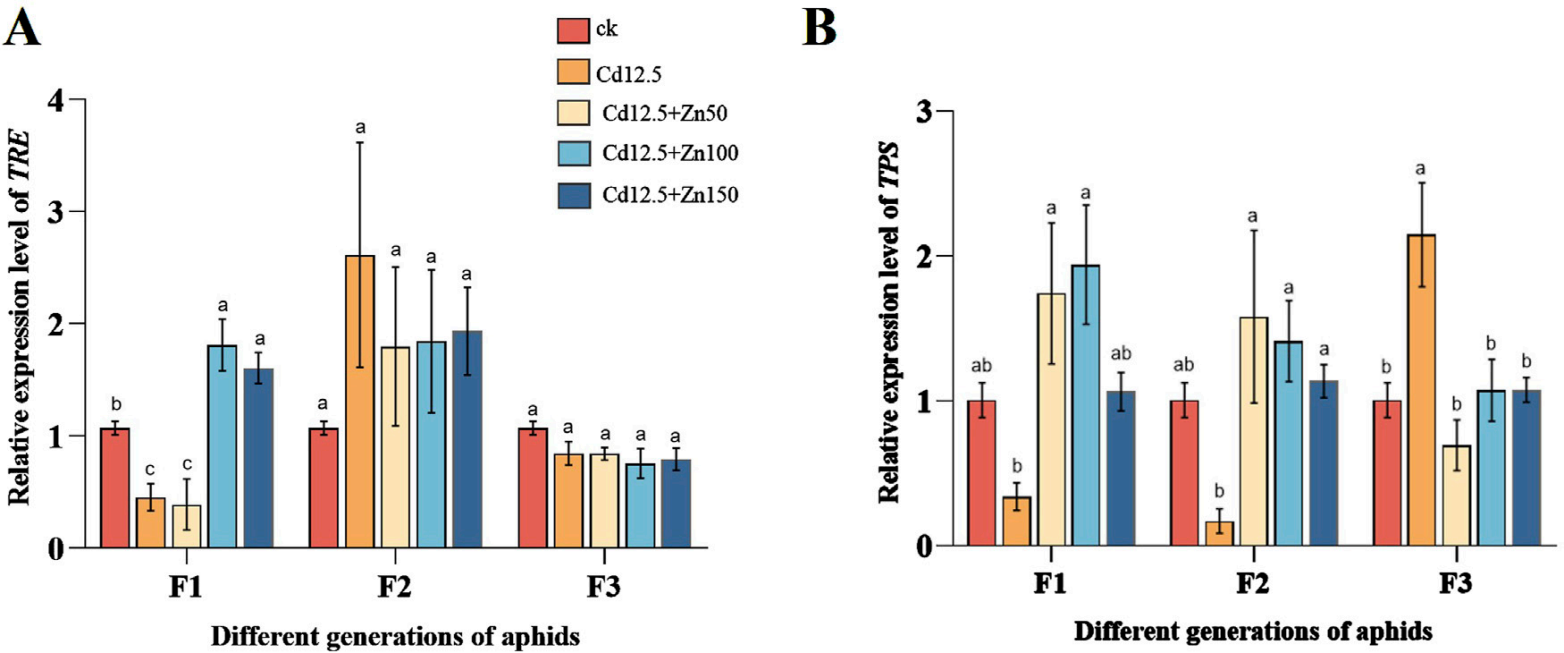
Changes in mRNA expression levels of *TRE* **(A)** and *TPS* **(B)** in different generations of aphids (F1, F2, and F3 represent three generations). McrActin expression level and RT-qPCR were used to calculate the relative expression level of target genes. The data are presented as the mean ± SE (n = 15 and N = 3). Bars with different letters indicate significant differences between different treatment groups of the same sample (Tukey’s method, *P* < 0.05).

In the F1 adult aphids, the relative expression level of *TRE* in the Cd12.5 mg/kg group was significantly lower than that in the control group, while the Cd12.5 + Zn100 mg/kg and Cd12.5 + Zn150 mg/kg groups showed significantly and extremely significantly higher expression levels, respectively, than the control. In the F2 and F3 adult aphids, there were significant differences in the relative expression levels of *TRE* in the Cd12.5 + Zn150 mg/kg and Cd12.5 + Zn50 mg/kg groups compared to the control group. Meanwhile, the relative expression levels of *TPS* in the F1 and F2 adult aphids in the Cd12.5 mg/kg group were significantly lower than those in the control. In the F1 adult aphids, the relative expression levels of *TPS* in the Cd12.5 + Zn100 mg/kg group were significantly higher than those in the control group, while in the F3 adult aphids, the relative expression levels of *TPS* in the Cd12.5 mg/kg group were significantly higher than those in the control group (Figure 5).

### 3.6 The number of offspring of female aphid and vitellogenin gene expression levels

**FIGURE 6:**
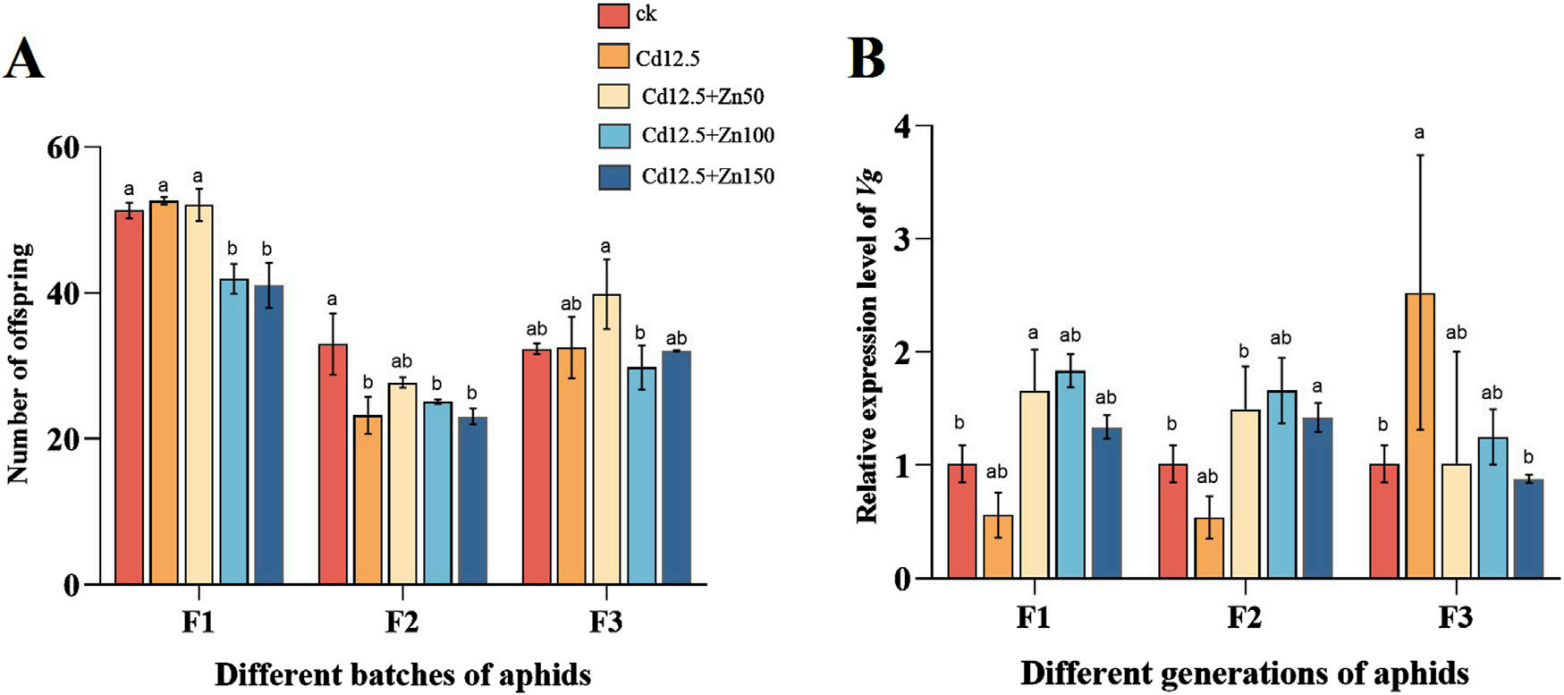
Postpartum analysis of single female aphids in different groups following different stress conditions **(A)**. The egg production of aphids in three generations changed (F1, F2, and F3 represent three generations). Changes in mRNA expression levels of *Vg* **(B)** in different generations of aphids. McrActin expression level and RT-qPCR were used to calculate the relative expression level of the target gene. The data are presented as the mean ± SE (n = 15 and N = 3). Bars with different letters indicate significant differences between different treatment groups of the same sample (Tukey’s method, *P* < 0.05).

F1 aphid production was significantly lower in the Cd12.5 + Zn100 mg/kg and Cd12.5 + Zn150 mg/kg groups than in the control group. In F2, except for the Cd12.5+Zn50 mg/kg group, the aphid production in the three treatment groups was significantly lower than that in the control group, while there was no significant difference between the groups in F3. In different groups of F1 female aphids, the expression level of *Vg* in the Cd12.5 + Zn50 mg/kg group was significantly higher than that in the control group, while in F2, the expression level of *Vg* in the Cd12.5 + Zn150 mg/kg group was significantly higher than that in the control group. In the F3 generation, the expression level of *Vg* in the Cd12.5 mg/kg group was significantly different from that in the control group (Figure 6).

## 4 Discussion

Soil heavy metal pollution has become a widespread issue, significantly impacting plants directly exposed to it and the animals that feed on these plants. After in-depth research, it was found that in reality, soil pollution mostly exists in the state of composite pollution of multiple heavy metals or coexists with other pollutants (Xing, 2018; Zhao et al., 2020). Cd and Zn, which belong to the IIB group of elements in the periodic table, are classified as thiophilic elements in the chemical classification of earth elements. In addition, due to their similar chemical properties, they are always found together in nature. Their presence in soil harms plants and then enters the terrestrial food chain, eventually reaching humans and posing a threat to human health (Wang et al., 2005). When multiple metal elements interact with each other, their composite effect significantly changes the original activity or toxicity of individual heavy metals, exhibiting antagonistic or synergistic promoting effects (Wang et al., 2018).

Heavy metal pollution can affect many aspects of plant growth, including seed germination, plant height, biomass, yield, and quality (Wang et al., 2023). Under Pb–Zn composite treatment, with the increase in the Zn concentration, the growth indicators of Taiwan Paulownia plants significantly decrease, the plant leaves turn yellow and wither, and the roots and stems cannot grow normally (Liu, 2013). In this study, under Cd–Zn-combined stress treatment, the germination rate of broad beans significantly decreased, and the higher the zinc concentration, the greater the decrease (Figure 1A). Within 9–15 days, a low concentration of heavy metal stress can increase seedling height, but high concentrations of Zn can hinder the growth of broad beans (Table 1). The decrease in these growth indicators is also consistent with the phenomenon of reduced plant biomass, as stated in previous studies (Liu, 2013; Pavithra et al., 2016; De Conti et al., 2020). Therefore, according to research (Wang et al., 2010), high concentrations of Zn disrupt the normal physiological and biochemical processes of root cells by inhibiting seed germination, lateral root formation, and affecting the luster of plant leaves, thereby inhibiting the growth and development of seedlings and leading to plant growth retardation.

The level of the chlorophyll content can reflect the photosynthetic capacity of plants, indicating the degree of leaf senescence and oxidation under environmental stress (Xia and Zhu, 2013). When the Zn concentration was 30 mg L^−1^, duckweed plants were damaged, 80% of the leaves lost their green color, and black spots appeared (Tang et al., 2010). In our experiment, there was also a phenomenon of decreased chlorophyll content under stress (Figure 1B). On one hand, it may be due to heavy metal stress that damages the structure of plant cell membranes and chloroplasts (Choudhury and Panda, 2005; Basile et al., 2012); on the other hand, it may affect chlorophyll synthesis by disrupting the enzymes required for chlorophyll synthesis, thereby inhibiting or even damaging the plant photosynthetic system (Li et al., 2012a). Alternatively, under high concentrations of heavy metal stress, Zn and Cd exhibit a synergistic effect, causing most leaves to lose their green color and chlorophyll function (Quan, 2022).

In both highland barley and wheat, the average contents of Cd and Zn varied in the order: root > leaf > stem (Tong et al., 2022). Our research results are consistent with these findings, and we found that the accumulation of Cd in broad beans is related to whether they are simultaneously infected with Zn (Figure 2). Zn can alleviate oxidative stress caused by Cd by stabilizing and protecting cell membranes, thereby slowing down the inhibition of Cd on rice growth (Hassan et al., 2005). In our study, when there was no aphid infestation, appropriately increasing the proportion of Zn under composite stress increased the Cd content in broad bean stems and leaves. Based on extensive research (Wang et al., 1999; Hassan et al., 2005; Liu et al., 2010; Li et al., 2012b; Rong et al., 2021), it is speculated that Zn weakens the damage caused by Cd in plants and stimulates the production of more carriers in roots, increasing the amount of Cd that can be fixed in plants. Due to transpiration, Cd is transported upward with water, gradually entering the upstream organs of plants and forming stable complexes. Feeding may alter the accumulation level of heavy metals in plants (Huang et al., 2021). We found that aphid infestation increases the Cd content in the roots of broad beans and the Zn content in the roots and stems but inhibits the increase in the Zn content under the leaves (Figure 2). This may be because aphid infestation leads to an increase in the concentration of Cd and Zn in plant phloem secretions (Stolpe et al., 2017a; Stolpe et al., 2017b), and the increase in the Zn concentration in soil makes Zn more competitive, allowing it to bind with zinc enzymes to maintain its original activity and prevent Cd toxicity (Wu, 2012), indirectly increasing the Cd content in roots. Otherwise, there may be another unidentified Cd absorption system in plant roots that involves systemic signaling (Plaza et al., 2015). Heavy metals can be transferred to phytophagous insects through food chains (Jiang et al., 2021), and during our research process, it was also found that aphids prefer to be densely distributed under leaves, resulting in a decrease in Cd and Zn content in the leaves.

Plant cells will accumulate a large number of osmotic regulatory substances such as proline and soluble sugar to reduce cell osmotic potential and regulate plant physiological metabolism under stress (Wu et al., 2019). Under Zn stress, the content of proline and soluble sugars in the roots and leaves of wheat seedlings significantly increased (Li et al., 2013). The content of proline in *Elytrigia elongata* significantly increased under the combined treatment of Cd and Zn (Tian et al., 2012). This study also found that Cd–Zn infection can significantly increase the soluble sugar content and proline content in the stems and leaves of broad beans (Figure 3). It is speculated that this is because plant cells secrete a large amount of stress-protective substances to alleviate heavy metal damage (Zhang et al., 2021). However, the accumulation of proline under Zn stress was not observed (Wan et al., 2023), and most reports on the role of proline are not consistent, highlighting the need for further study (Tanwir et al., 2021).

Trehalose, as an energy substance and structural component, can protect organisms from environmental stress under adverse conditions and play an important role in cellular energy metabolism and other processes (Thompson, 2003; Zhao et al., 2021). Trehalase belongs to glucosidase, which can catalyze the hydrolysis of one trehalose molecule to produce two glucose molecules, and it is widely present in microorganisms, insects, and plants (Shukla et al., 2015). Membrane-bound trehalase mainly exists in the muscles, while soluble trehalase mainly exists in the small intestine and brain tissue (Yang et al., 2007). In our study, under heavy metal infection, the activity of soluble trehalase in F1 and F2 adult aphids was significantly enhanced, while membrane-bound trehalase showed no significant change, resulting in a significant decrease in the trehalose content, but the phenomenon exhibited by F3 adult aphids was the opposite (Figures 4A, D, E). This may be because when aphids were initially infected with heavy metals, they obtained glucose by hydrolyzing trehalose (Figure 4B), which helped protect somatic cells and allowed them to better adapt to stress environments (Behm, 1997). After three generations, aphids gradually adapted to heavy metal environments, relying solely on glucose metabolism generated by glycogen breakdown to meet their life activities (Figure 4C). *TRE* and *TPS* are trehalase and trehalose synthase genes in insects, playing important roles in their physiological activities. In a dry environment, the expression of *TPS* in *Drosophila melanogaster* is enhanced, the expression of *TRE* is weakened, and the content of trehalose in the body is increased (Thorat et al., 2012). In our experiment, the F3 generation under heavy metal stress conforms to this phenomenon, while the expression of *TPS* and *TRE* is enhanced in F1 and F2 and is more significant when the Zn content is 100 mg/kg (Figure 5). It is speculated that this is because under external stimulation, trehalose metabolism in aphids is vigorous, and enzyme gene expression is also feedback-regulated (Chen et al., 2017).

Insect Vg and YP are the two main types of yolk proteins in insects, and Vg is a precursor substance of YP, which can be degraded into small-molecule peptides and amino acids during insect embryogenesis, providing nutrients and functional substances such as amino acids, fats, and carbohydrates for embryonic development (Tufail and Takeda, 2009; Yan et al., 2010). However, recent studies suggested that *Vg* plays an important role in other aspects of insect biology, such as the caste differentiation process in social insects, wound healing, protection against oxidative stress, immunity, and life span regulation (Kodrík et al., 2019). In addition, clarifying the important egg-laying-related gene *Vg* and its regulatory mechanism can provide new ideas for the prevention and control of *Hemiptera* insects (such as aphids), which is of great significance for effectively reducing agricultural and forestry losses (Pan et al., 2023). In our study, F1 aphids significantly accumulated trehalose under Cd–Zn-mixed stress (Figure 4A), while female aphid production showed a significant decrease (Figure 6A), which is consistent with the inference that trehalose accumulation, which usually occurs in stress responses, diminishes reproductive activity and reduces longevity (Zhou et al., 2022; Xu et al., 2023). However, partial Cd–Zn composite treatment increased the expression level of *Vg* (Figure 6B), which is not completely consistent with the reduction in female aphid production. It is speculated that aphids can achieve stress protection by expressing Vg, which is consistent with the inference that this gene has potentially acquired non-reproductive functions (Cardoso-Júnior et al., 2021). Therefore, it can be speculated that heavy metal stress, to some extent, inhibits female aphid production, but it may not be indirectly inhibited by affecting *Vg* expression. Instead, it may reduce production by regulating insect trehalose accumulation and inhibiting reproductive activity. This requires further exploration to determine whether there are other pathways of influence and regulatory mechanisms.

## 5 Conclusion

In summary, the results showed that Cd (12.5 mg/kg) and different levels of Cd–Zn-combined treatment promoted the growth of broad beans, increasing seedling height, but when the Zn concentration was too high, a synergistic inhibitory effect was observed. The absorption capacity and distribution of heavy metals in various components of broad beans varied, resulting in differences in the content of heavy metals in different parts. Moreover, under Cd–Zn composite treatment, aphids infesting the plants promoted the absorption of Zn through the roots and stems of broad beans. In addition, during Cd–Zn composite treatment, broad beans adapted to heavy metal complex stress by regulating total soluble sugar, proline, and chlorophyll metabolism in each plant component. Meanwhile, with intergenerational changes, different treatment groups dominate the expression levels of *TRE* and *TPS* genes in each generation. Correspondingly, the sugar content and trehalase activity in aphids respond to the expression. The number of offspring of female aphids in each group of three generations showed a decreasing trend initially, followed by an increase, with intergenerational changes, and the trend of Vg expression levels was slightly different, further confirming the close relationship between insect trehalose metabolism and reproduction, as well as the possible role of Vg in other life activities of insects. These findings suggest the need for further experiments to explore these mechanisms.

## Data Availability

The datasets presented in this study can be found in online repositories. The names of the repository/repositories and accession number(s) can be found in the article/Supplementary Material.
