# Supplementary material for: Effect of Cd–Zn compound contamination on the physiological response of broad bean and aphids
Source: Front Physiol. 2025 Feb 5;16:1533241. doi: 10.3389/fphys.2025.1533241 (PMC11835991; doi:10.3389/fphys.2025.1533241)
Supplement: Supplementary file 1 [file Table1.DOCX]

## Figures and tables caption

Table S1 Primers used for quantitative real-time PCR

| Gene Name | Primer Name | Nucleotide Sequences (5' -3') |
| --- | --- | --- |
| *Vg* | Mc*Vg*-F | GCATTAGCCACTATGTTTCA |
|  | Mc*Vg*-R | CGTATTGCTCCATTGTTGT |
| *TPS* | Mc*TPS*-F | CGTGGACAGGCTAGACTACA |
|  | Mc*TPS*-R | CAGCTCAGTCTCGTCCTTGA |
| *TRE* | Mc*TRE*-F | TGGCAAGATACTACGCACCA |
|  | Mc*TRE*-R | ATCAGCCAATACCCCACGAT |
